# Polarized SERS Controlled by Anisotropic Growth on Ordered Curvature Substrate

**DOI:** 10.3390/molecules26082338

**Published:** 2021-04-17

**Authors:** Yaxin Wang, Aonan Zhu, Xiaolong Zhang, Yongjun Zhang

**Affiliations:** 1 Center for Advanced Optoelectronic Materials, School of Material and Environmental Engineering, Hangzhou Dianzi University, Hangzhou 310018, China; yaxinwang@hdu.edu.cn; 2 College of Chemistry, Nankai University, Tianjin 300071, China; aonanzhu@mail.nankai.edu.cn; 3 College of Physics, Jilin Normal University, Changchun 130103, China

**Keywords:** angle deposition, polarization, SERS

## Abstract

Colloidal lithography is an efficient and low-cost method to prepare an ordered nanostructure array with new shapes and properties. In this study, square-shaped and cone-shaped Au nanostructures were obtained by 70° angle deposition onto polystyrene bead array with the diameter of 500 nm when a space of 120 nm is created between the neighbor beads by plasma etching. The gaps between the units decrease when the Au deposition time increases, which leads to the polarized enhanced local field, in agreement with the surface-enhanced Raman scattering spectra (SERS) observations and finite-difference time-domain (FDTD) simulations. When the Au deposition time increased to 5 min, 5 nm gaps form between the neighbor units, which gave an enhancement factor of 5 × 10^9^. The SERS chip was decorated for the detection of the liver cancer cell marker Alpha-fetoprotein (AFP) with the detection limit down to 5 pg/mL.

## 1. Introduction

Plasmonics is one of the important research fields with the focus on the interactions among photons, electrons, and metal nanostructures, in which many interesting phenomena are observed due to the regulated collective movements of the electrons excited by the incident light, such as the abnormal transmission of light [1,2,3], surface-enhanced spectroscopy [4,5], and localized heating on the nanometer scale, and so on [6]. Among these observations, the surrounding media, the chemical compositions and microstructures, and the nanostructure patterns play very important roles in the properties [7,8]. To drive the light to go along the expected route, the dielectrics and composition distributions of the surrounding conditions are designed by Zhu’s group, and the interaction of the media materials confines the light movement in a certain area like a black hole [9]. When the metal nanostructures are prepared as the core coated by other materials, the surface plasmons can be confined around the core by the coverage layer, which tends to work as the controllable hotspot for high-sensitivity detections and is used in various fields for detection [10,11,12,13]. In addition to the composition and the surrounding conditions, the nanostructures with different shapes show some exciting properties and applications. To investigate the interesting behaviors of electrons and photons in metal nanostructures, the various shapes of metal nanostructures are designed in an ordered pattern, which are proposed to regulate the electron movements and the interactions [14,15,16,17]. In fact, the polarization effect caused by the anisotropic shape finds very important applications in plasmonic sensors for the self-reference effects [18,19]. In these patterned materials, the metal units give contributions to the enhanced local field besides the gaps, owing to the coupling surface plasmon excited by the light, which depends on the angle between the polarization direction of the electric field and the normal direction of the gap [20]. Among these techniques, nanosphere lithography is a simple process for ordered nanostructure fabrication, based on how many new nanostructures are fabricated, compared to conventional lithography. In previous works, we realized the fabrication of a nanobowl array, nanohoneycomb, and nanorod array, where some interesting applications were obtained, such as surface-enhanced Raman scattering spectra (SERS), surface-plasmon-assisted nanostructure growth [21]. In this work, an angled deposition was performed onto two-dimensional polystyrene (PS) beads array. By controlling the distance between the PS beads and the deposition angle and the film thickness, the square-shaped Au and peach-shaped Au nanostructures were obtained, which showed significant polarization properties in transmission spectra and SERS observations, which was possible to enhance the detection sensitivity.

## 2. Materials and Methods

The solution 10 wt% of polystyrene (PS) beads with a size of 500 nm were obtained from Huake Weike, Wuhan, China. Si wafers (100) were purchased from Hefei Kejing Co., Ltd. of Materials Technology, Hefei, China. Sodium lauryl sulfate, 4-mercaptobenzoic acid (4-MBA), ethanol, NH_3_·H_2_O, tetrahydrofuran, N-Hydroxysuccinimide (NHS),1-(3-Dimethylaminopropyl)-3-ethylcarbodiimide hydrochloride (EDC), and H_2_O_2_ were bought from Aladdin Co., Ltd., Beijing, China. Au targets purity was 99.99%, supplied by Beijing TIANQI Co., Beiing, China. The deionized water of 18.0 MΩ cm^−1^ was prepared in our laboratory. AgNO_3_ and C_6_H_5_-Na_3_O_7_·2H_2_O were used as supplied. The silicon substrates with the size 1 × 1 cm^2^ were cleaned in a boiling solution (NH_4_·OH, H_2_O_2_, H_2_O = 1:2:6) for 5 min and then cleaned ultrasonically in ethanol for 5 min. The mixture of PS and ethanol was prepared with a volume ratio of 1:1. The Si substrate, covered by the mixture of PS and ethanol, was dipped into water, which resulted in a floating PS monolayer film on the water surface. PS bead size was decreased by plasma etching (Fischione model 1020) under the power of 20 W, with the mixture gas of 80% Ar and 20% O_2_ in volume. The PS monolayer on Si substrate was used chosen for Au deposition in a magnetron control system (Shenkeyi company, Shenyang, China) with a sputtering power of 25 W and a pressure of 0.5 Pa. The Au structures were immersed in a 4-MBA solution (10^−3^ mol/L in ethanol) for 30 min and then incubated in a mixture of NHS/EDC for 120 min, followed by deionized water washing and nitrogen drying. Further, the nonspecific binding was minimized by incubation of the chip with a blocking buffer for 2 h. The SERS-active substrates further modified the anti-Alpha-fetoprotein (AFP) species (20 μL anti-AFP, 37 °C, 4 h). Scanning electron microscopy (SEM) images were obtained by a JEOL 6500F field emission scanning electron microscope (15 kV). Raman measurement was recorded on a Renishaw Raman system model 2000 confocal microscopy spectrometer (633 nm, 20 mW). The objective magnification was 50× long-range objective, and the laser spot size was 1 μm in diameter. The finite-difference time-domain (FDTD) was used to simulate the hotspots distribution pattern in the nanostructure. The polarized light axes were used as excitation light. According to the periodicity of the structure, the periodic boundary and perfectly matched layer boundary conditions were applied to the xy-plane and z-direction. The grid accuracy was set to 2 nm due to the limited availability of computer resources. The auto-shutoff minimum was fixed to 1 × 10^−5^, and the overall simulation time to 1000 s. The simulation temperature was 300 K. The fitting parameters of Au material were obtained from the material library of FDTD.

## 3. Results and Discussion

**Figure 1:**
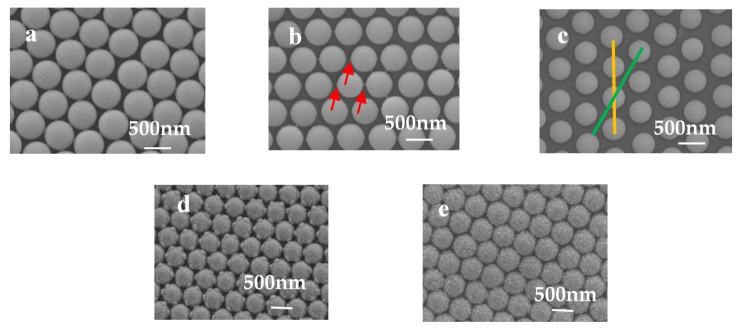
(**a**) Polystyrene (PS) 500 nm array by self-assembly and after etching for 3 min (**b**) and vertical deposition of Au film (**d**); (**c**) PS500 nm array by self-assembly after etching for 3.5 min and vertical deposition of Au film (**e**).

The sizes of the PS beads were decreased, obviously, by the plasma etching process, as shown in Figure 1. When the plasma etching process was applied to the 2D PS array for different times, the PS array of the different morphologies were obtained, due to the shadow effects from the neighbor PS beads, which means that the decreased etching rate neared the touching parts. When the etching time was 3 min for the 500 nm PS array, small protruding parts were observed around the PS array, as shown by the red arrows, due to the shadow effects from the neighbor PS beads, as reported in previous works [22]. When the etching time was over 3 min, for example, 3.5 min, the protruding parts were etched away, and the PS beads showed the smooth surfaces again.

When Au film is deposited onto the PS array vertically, that is, along the normal direction, the Au nanocaps form on the PS array. For the PS etched for 3 min, the Au nanocaps showed the protruding parts due to the fast growth rate of Au film around the protruding parts induced by the large local strain, as shown in Figure 1d. For the PS array etched for 3.5 min, the Au nanocaps showed uniform growth along the PS surface, and no protruding parts were observed, as shown in Figure 1e. To get the nanostructures of the different shapes, the angle deposition was applied, which means the deposition deviated from the vertical direction, as shown in Figure S1. Generally, the curved surfaces induced a fast growth along the film deposition direction and a slow lateral growth, which led to the formation of nanorods for long-time depositions, as reported due to the preferential growth [23]. Although many hotspots were prepared between these nanorods, the beneath hotspots showed tiny contributions to the total SERS signals because they were far away from the sample surface and could not be detected. Therefore, the hotspots near the sample surface were preferred in practical applications. When the 500 nm PS array was etched for 3.5 min, the neighbor PS beads were separated completely, and the gaps between the neighbors were around 120 nm. Then the deposition angle was set as 70°, and the Au deposition was carried out.

**Figure 2:**
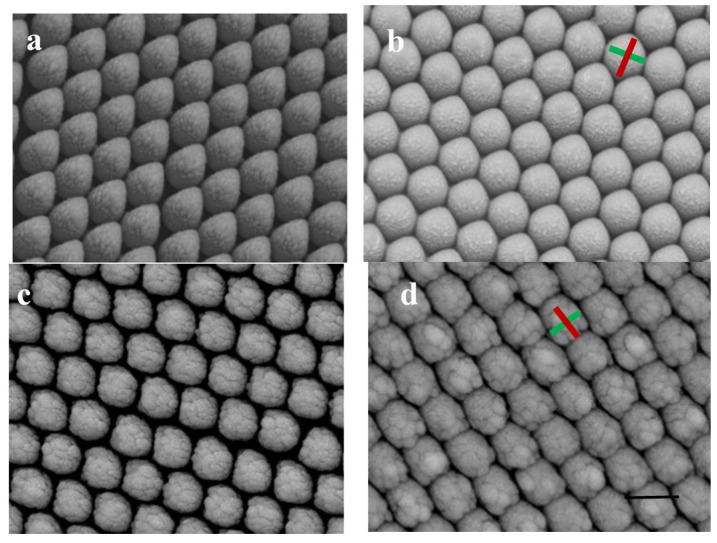
Square- and cone-shaped Au nanostructures on PS500 nm array by 70° angle deposition for 10 min (**a**,**c**) and 15 min (**b**,**d**). The scale bar is 500 nm for all images. P polarization means the E vector of exciting light is along the red line. S polarization means the E vector of exciting light is along the green line.

During this deposition process, there were two kinds of possible nanostructures obtained on the PS array. One was the nanostructures of a square shape, and the other was a cone shape, as shown in Figure 2. When the deposition was along the green line, as shown in Figure 1b the film growth rate was fast along the deposition direction because the distance to the shadowing neighbor was very large, and the shadow effect was reduced greatly. Therefore, the film grew fast compared to the lateral growth. The lateral size was 400 nm, and the longitudinal size was 510 nm when the deposition time was 10 min, which led to the formation of the cone-shaped film, as shown in Figure 2a. When the film deposition continued to 15 min, the longitudinal size was measured as 510 nm, and the lateral size was 500 nm. The lateral growth decreased the gap sizes between the neighbor cones, which increased the shadow effects from the neighbors, and the longitudinal growth rate was reduced significantly. In comparison, the lateral growth increased, resulting in the structures of the hexagon-like shapes, as shown in Figure 2b. When the deposition was along the yellow line, as shown in Figure 1b, the film growth rates showed small differences between the deposition direction and the lateral direction because the similar shadow effects came from the shadowing neighbor on the right side, the left side, and the front side, as shown in Figure S2. When the deposition time was 10 min, the lateral size was 470 nm, and the longitudinal size was 480 nm along the deposition direction, as shown in Figure 2c, which means the shadow effects were similar around for this condition. For each Au cap, the front part also showed the decreased curvature of the surface due to the reduced shadow effect caused by the enlarged distances along the bead surface, which indicated that long-time growth led to a flat front surface. For the back part of Au caps, the self-shadow effect made the nanocap grow into a square-like shape, which means that the large distance led to fast growth and the small distance led to slow growth. The self-shadow effect led to square-shape nanostructures when the deposition time was 15 min, as shown in Figure 2d.

**Figure 3:**
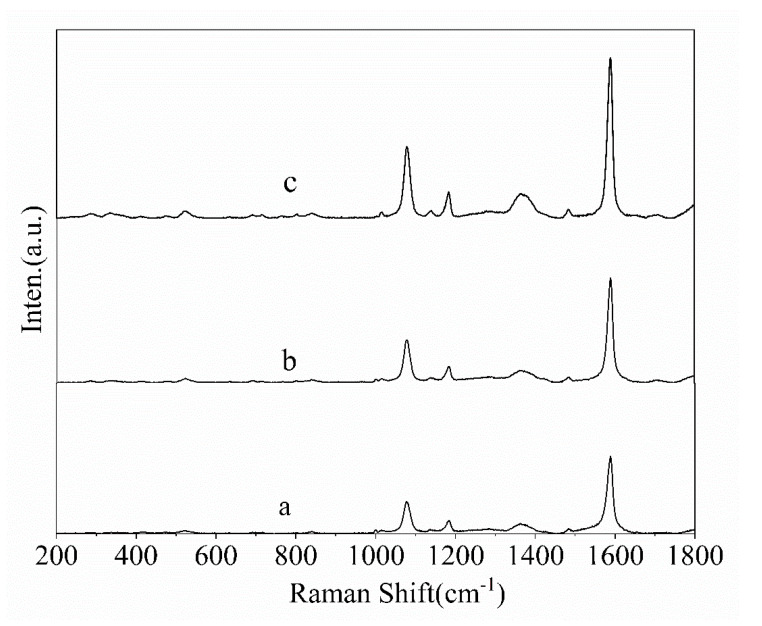
In comparison with Au nanocap (**a**) and Au cone array (**b**), Au square array (**c**) shows excellent surface-enhanced Raman scattering spectra (SERS) performance.

**Figure 4:**
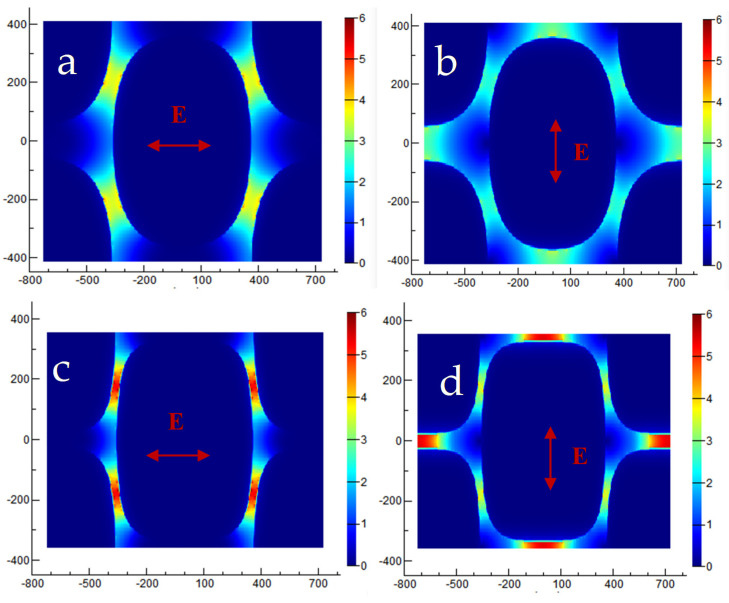
Finite-difference time-domain (FDTD) simulation shows that p polarization gives the significant enhanced coupling electric field vector E due to the narrow gaps in square-shaped Au nanostructures with the deposition time of (**a**,**b**) 10 min and (**c**,**d**) 15 min. The scale units of x and y are in nanometer for both images.

4-MBA (10^−3^ M) was used as probe molecules to evaluate the potential effect of the nanostructures as SERS-active substrates, Au square array, and Au cone array with deposition of 15 min. Calculation of the enhancement factor (EF) is performed as EF = (I_SERS_/N_SERS_)/(I_bulk_/N_bulk_), where I_SERS_ and I_bulk_ are the SERS intensities of 4-MBA on the samples and normal Raman scattering intensity of the solid sample of 4-MBA, respectively. N_bulk_ and N_SERS_ are the numbers of the 4-MBA molecule adsorbed on the samples and bulk molecule illuminated by the laser excitation to obtain the corresponding SERS and normal Raman spectra, respectively. In fact, EF means the ratio of the SERS signals to Raman signals for every single molecule on even. In our experiment, the laser spot was 1 μm in diameter and the penetration depth was 17 μm of the focused laser beam used; the density of 4-MBA was 1.345 g/cm^−3^; thus, the number of the 4-MBA molecule illuminated by the laser light was calculated to be 7.01 × 10^10^. N_SERS_ was the number of surface-adsorbed molecules within the laser spot with the diameter (1 μm), and the surface area occupied by an adsorbed molecule could be adopted as 0.20 nm^2^/molecule. In comparison with the deposition along the normal direction, both Au square array and Au cone array showed an enhancement factor of 5 × 10^9^ and 3 × 10^9^ for the p polarization light, as shown in Figure 2, indicating the Au square array with excellent SERS performance due to the narrow gaps (Figure 3). FDTD simulation was used to demonstrate the hotspot distribution in the Au square array structure, which proved the local coupling increased with the Au deposition of time when the gaps decreased between the neighbor units. In comparison with other nanostructures based on PS templates, the hotspot distributions in our observations were like those from the squared-shape noble nanostructures fabricated by conventional lithography with more hotspot distributions, as shown in Figure 4. When the deposition time increased from 10 min to 15 min, the shapes of the nanostructures were quite like the squared, and more hotspots were created. In addition, the local electromagnetic fields increased significantly due to the narrow gaps between the neighbor units when the deposition time increased. Compared to s polarization, p polarization resulted in more hotspots; therefore, the nanostructures by 15 min deposition showed the larger enhancement factor. FDTD simulations also indicated the SERS properties for Au deposition with the angle of 70° showed the different symmetries and hotspot distributions affected by the template pattern and the film deposition parameters, indicating more possibilities to control the morphologies of the nanostructure array. Similar observations were also obtained for the cone-shaped samples, as shown in Figure S3.

**Figure 5:**
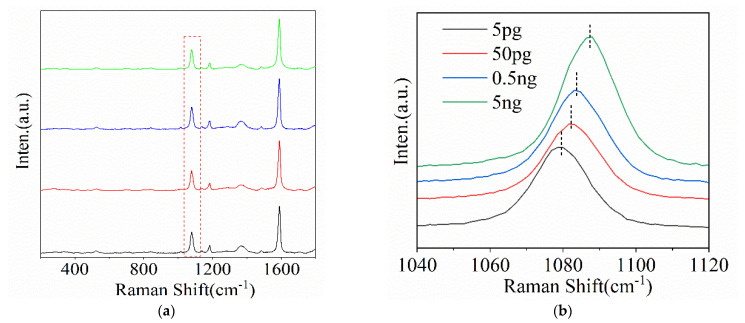
(**a**) AFP concentration from 5 pg to 5 ng/mL results in a continuous shift of the peak at 1075 cm^−1^. (**b**) shows the enlarged image of the peak around 1075 cm^−1^.

The nanostructure with the square shapes was used as the SERS-active substrate for detecting AFP due to its excellent SERS when excited by p-polarization light. After 4-MBA decoration, the EDC-NHS mixed solution was applied to activate the carboxyl group for the generation of MBA-derived coupling agent, forming the SERS-active chips [24]. To avoid non-specific recognition after the anti-AFP modification, the chip was deactivated with a blocking buffer, which was to clear the cross-link active sites and avoid the reaction with any other proteins. SERS-active chips were immersed in AFP solutions of different concentrations for specific and saturated recognition. As reported, the frequency shift of the peak at 1075 cm^−1^ belonged to the ph-S-Au vibration mode, which could be used for quantitative analyses of the AFP concentration because the peak at 1075 cm^−1^ showed consistent shifts with AFP linkage amount [25]. For our sample with square-shaped units, the excitation light with p-polarization showed the significant SERS observations due to the significant hotspot distribution, and the peak at 1075 cm^−1^ showed continuous shifts when the AFP concentration increased from 5 pg to 5 ng/mL, confirmed by the magnified peak at 1075 cm^−1^, as shown in Figure 5. Similar observations were also reached for the sample with the cone-shaped units. When the p polarization light was applied, the mean enhanced electromagnetic field was also very strong in comparison with the s polarization light, which means that both structures were suitable for AFP detection. These observations indicated that our preparation method was simple for good SERS signals based on 2D PS beads arrays.

## 4. Conclusions

Based on the 2D PS array template, Au arrays with different shapes were prepared by angle deposition. When the deposition angle was 70°, the square-shaped and cone-shaped nanostructures were obtained on PS500 nm with a space of 120 nm after being etched. The increase in Au deposition time led to the Au caps showing a cube shape due to the similar shadow effects around. At the same time, the increased time of Au deposition results in the narrow gaps between Au caps, which made significant contributions to the enhanced local coupling electromagnetic field, was confirmed by significant SERS observations and FDTD simulations. The SERS chip was made for detecting the liver cancer cell marker AFP, and the detection limit of AFP in the range of 5 pg to 5 ng/mL was obtained, which provided a possible early detection of liver cancer.

## Data Availability

The data presented in this study are available on the request from the corresponding author.

## Supplementary Materials

The following are available online, Figure S1: deposition setting-up, Figure S2: different directions of the film depositions onto the PS array, Figure S3 and Figure S4: different directions of the film depositions leading to the nanostructures with different shapes, Figure S5: exciting light with P and S polarizations.

## Sample Availability

Samples of the compounds Not available from the authors.
