# Supplementary material for: Polarized SERS Controlled by Anisotropic Growth on Ordered Curvature Substrate
Source: Molecules. 2021 Apr 17;26(8):2338. doi: 10.3390/molecules26082338 (PMC8073224; doi:10.3390/molecules26082338)
Supplement: Supplementary file 1 [file molecules-26-02338-s001.zip › molecules-1118039-supplementary.pdf]

Polarized SERS Controlled by Anisotropic Growth on Ordered Curved Substrate

Y.X. Wang[1,2], A.N. Zhu[3], X.L. Zhang[4*], Y.J. Zhang[1,2*]

[1]School of Material and Environmental Engineering, Hangzhou Dianzi University, Hangzhou 310018, P.R. China.

[2] Research Center for Advanced Optoelectronics, School of Material and Environmental Engineering, Hangzhou Dianzi University, Hangzhou 310018, P.R. China

[3] College of Chemistry, Nankai University, Tianjin 300071, P.R. China.

[4] College of Physics, Jilin Normal University, Changchun 130103, PR China

* Correspondence: yjzhang@hdu.edu.cn

Deposition angle means the angle between the normal direction of the substrate and the vertical direction.

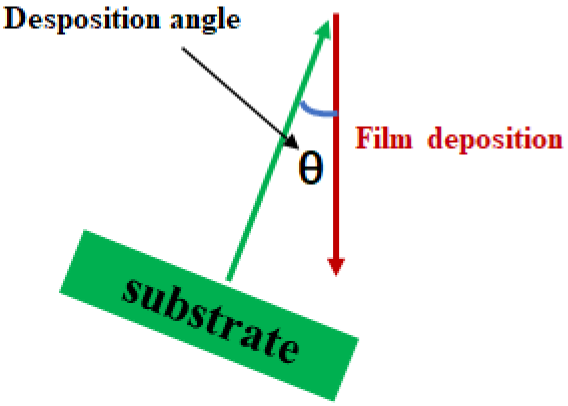

Figure S1

The yellow line and the green line show two different directions of the film depositions, chosen in our work.

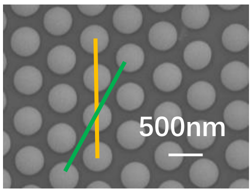

Figure S2

When the deposition angle θ is 70 degrees and the deposition direction is along the green line, the cone-shaped nanostructures form on the ordered PS500nm array etched 3.5min.

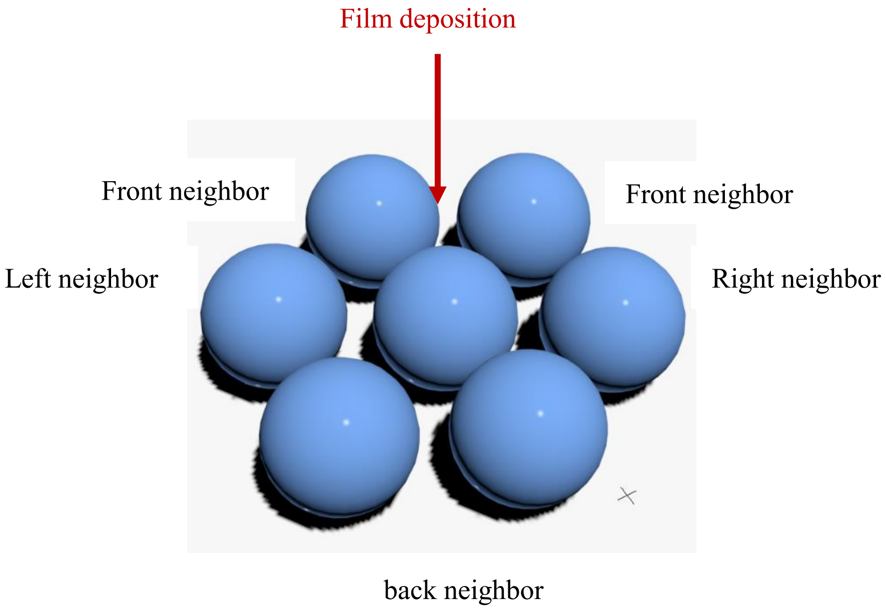

Figure S3

Due to the shadow effects from the neighbors and self-effect, the film depositions onto each PS bead show different growth mechanisms. When 70 degrees deposition is performed, the square-shaped unit is obtained.

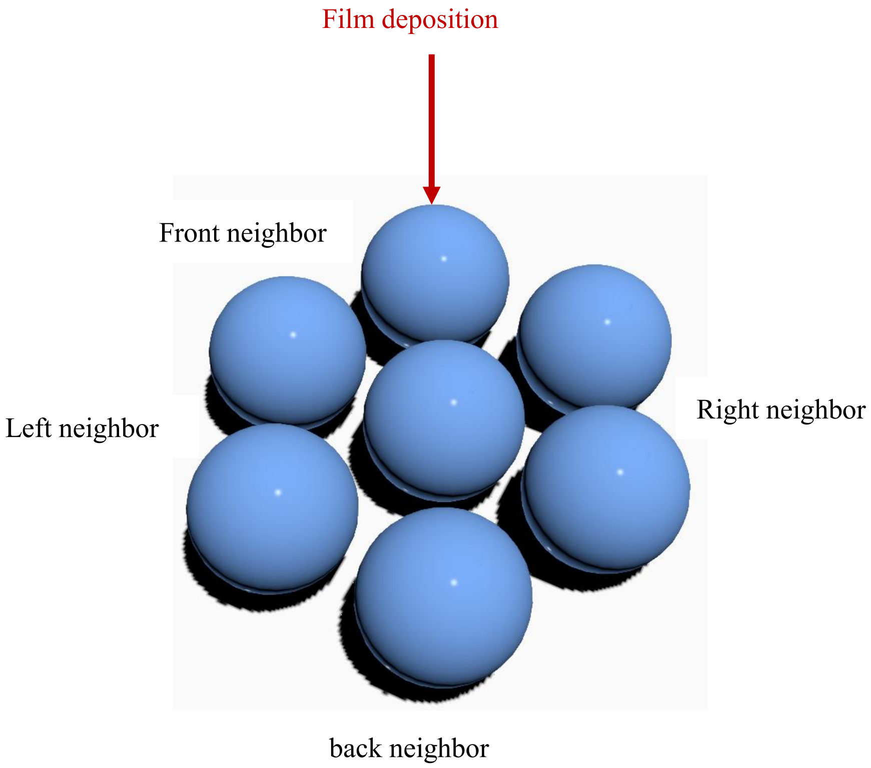

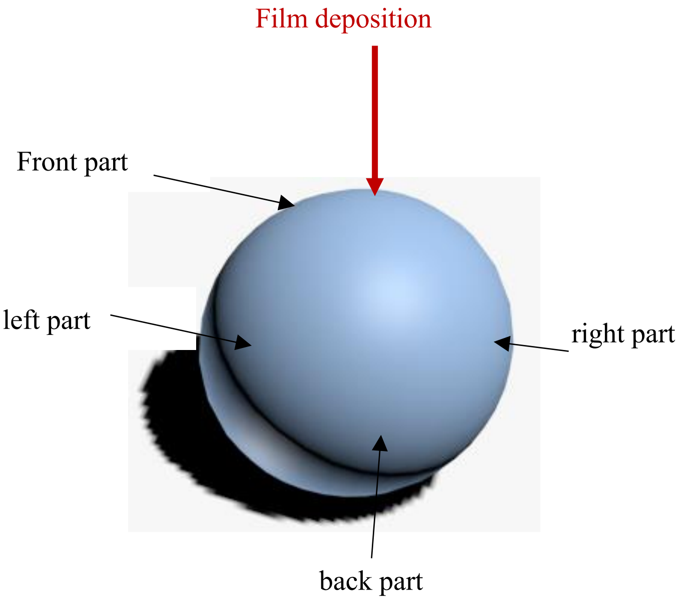

Figure S4

P polarization means the E vector of exciting light is along the red line. S polarization means the E vector of exciting light is along the green line.

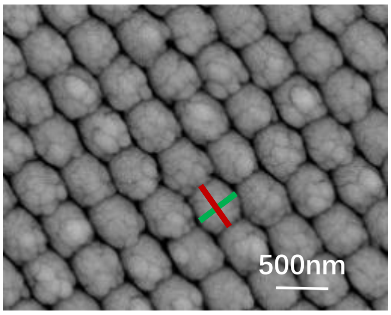

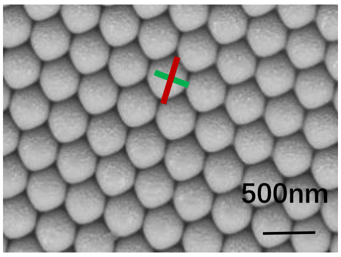

Figure S5

FDTD simulations show the square-shaped show better SERS properties compared to the cone-shaped. And the improved SERS properties are from the narrow gaps and more hotspots when excited by p polarized light. Long time deposition decreases the gaps between the neighbors, leading to the stronger coupling.

Square shape 10min 15min

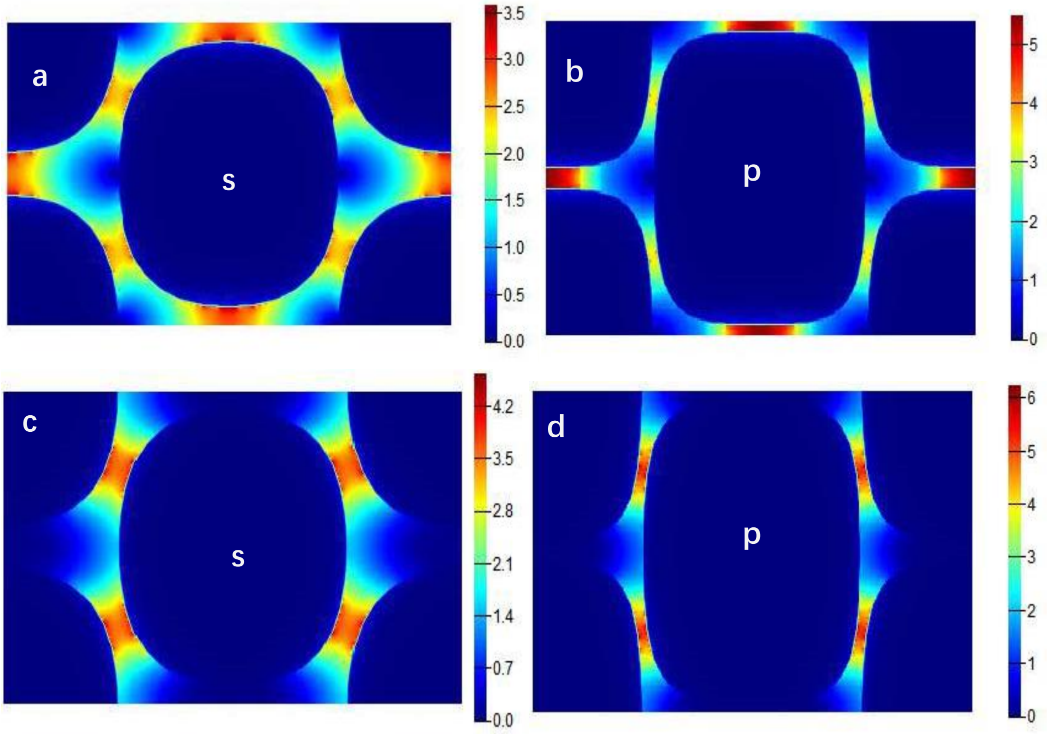

Cone shape

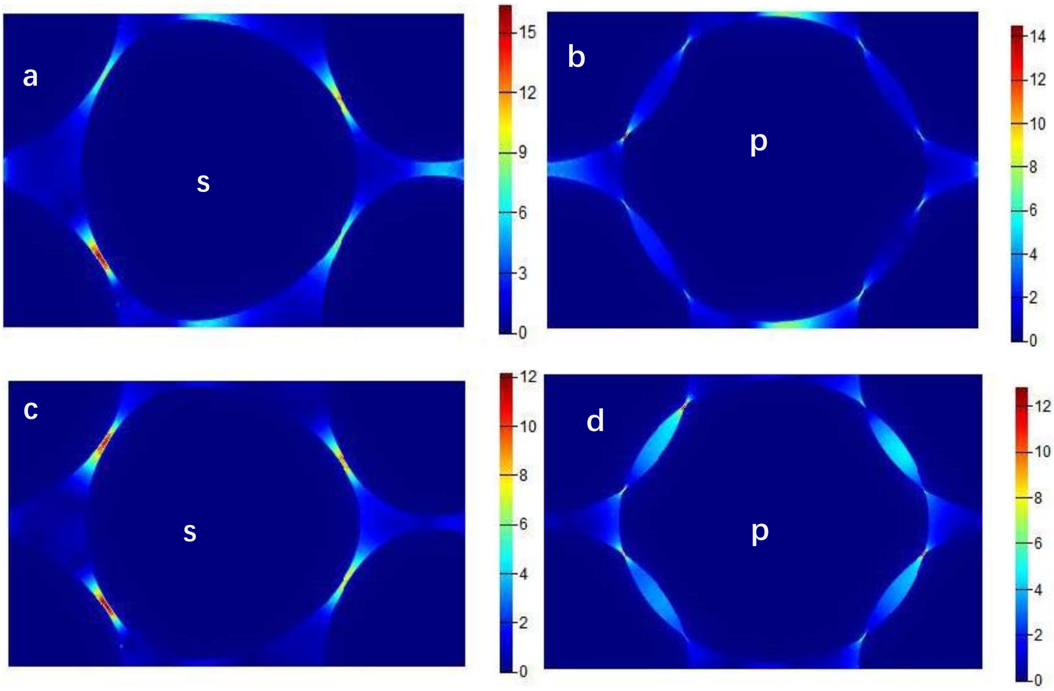

Figure S6
